# Prevalence of Sarcopenia and Factors Associated with Body Composition and Protein Intake in Institutionalized Older Adults from Three Long-Term Care Facilities in Loja, Ecuador: A Cross-Sectional Study

**DOI:** 10.3390/geriatrics11050115

**Published:** 2026-08-31

**Authors:** Ana Sofía Vivanco-Zárate, Mateo Julián Sánchez-Sánchez, Estefanía Bautista-Valarezo

**Affiliations:** 1 Nutrition and Dietetics Program, Faculty of Health Sciences, Universidad Técnica Particular de Loja, Calle París, San Cayetano Alto, Loja 110101, Ecuador; mjsanchez47@utpl.edu.ec; 2 Medicine Program, Faculty of Health Sciences, Universidad Técnica Particular de Loja, Calle París, San Cayetano Alto, Loja 110101, Ecuador; mebautista@utpl.edu.ec

**Keywords:** sarcopenia, institutionalized older adults, body composition, protein intake, nutritional status

## Abstract

**Background/Objectives:** Sarcopenia is a common public health problem in older adults and is associated with an increased risk of morbidity and mortality, particularly in institutionalized populations. This study aimed to determine the prevalence of sarcopenia in institutionalized older adults, assess the adequacy of protein intake in terms of quantity and quality, and identify factors associated with its presence. **Methods:** An observational, analytical, cross-sectional study was conducted in three institutional care homes in Loja canton, Ecuador, between 2024 and 2025. A total of 44 older adults aged ≥65 years were included. Sarcopenia was diagnosed according to the EWGSOP2 criteria, and protein intake was assessed using the direct food-weighing method over three days. Descriptive, comparative, and binary logistic regression analyses were performed. **Results:** The prevalence of confirmed and severe sarcopenia was 61.3%. Severe sarcopenia was more frequent in men, whereas probable sarcopenia was more frequent in women. Participants with sarcopenia presented with lower skeletal muscle mass, skeletal muscle index, and muscle strength. Protein intake was generally insufficient compared with international recommendations, although protein quality was predominantly high or very high. Higher skeletal muscle mass and higher body mass index were independently associated with a lower likelihood of sarcopenia. **Conclusions:** Sarcopenia in institutionalized older adults appears to be more closely associated with body composition and overall nutritional status than with protein quantity or quality alone. These findings support a comprehensive approach to the prevention and management of sarcopenia that combines adequate nutrition with tailored physical exercise interventions.

## 1. Introduction

Sarcopenia is a condition characterized by the progressive and generalized loss of skeletal muscle mass, accompanied by a decline in muscle strength and/or physical performance. Its prevalence varies depending on the diagnostic criteria and healthcare setting used for its assessment [1]. Globally, the prevalence of sarcopenia among older adults is estimated to range between 10% and 16% [2]. In Latin America, the prevalence reaches approximately 13.33%, with a higher burden observed in women. In Ecuador, a higher prevalence has been reported among adults aged 70 to 75 years, with a more pronounced impact in women over 80 years, who are present an incidence of 40% compared to 25% in men [3].

Population aging has positioned sarcopenia as a significant public health concern, as it is associated with increased morbidity and mortality [4]. Among community-dwelling older adults, low self-reported protein intake and low levels of physical activity have been linked to a higher risk of sarcopenia. Individuals with a protein intake ≤ 1 g/kg/day and physical activity levels ≤ 150 min/week tend to present with lower muscle mass, higher fat mass, and poorer performance in strength and physical function [5].

Current evidence indicates that adequate nutrition enhances the beneficial effects of exercise on muscle mass and function, supporting the combined use of nutritional strategies and physical activity for the prevention and management of sarcopenia [6,7]. In the absence of approved pharmacological treatments, current guidelines prioritize lifestyle-based interventions, particularly emphasizing protein intake. A minimum intake of 1.0 g/kg/day is recommended, although some authors suggest a range of 1.0–1.2 g/kg/day [5,8].

International expert groups recommend a Population Reference Intake (PRI) of 1.0–1.2 g/kg/day for healthy older adults, increasing to 1.2–1.5 g/kg/day in the presence of acute or chronic disease, and up to 2.0 g/kg/day in cases of severe illness, injury, or malnutrition. In addition to total daily intake, the distribution of protein consumption is also critical, as at least 0.4 g/kg per meal is required to optimize postprandial muscle protein synthesis at rest [9].

Despite the growing evidence, studies focusing on institutionalized older adults remain limited, particularly in Latin American countries such as Ecuador. Institutionalization has been associated with higher mortality, poorer quality of life, social isolation, malnutrition, chronic diseases, and increased functional dependence [10], which may hinder the implementation of preventive strategies. Furthermore, evidence simultaneously evaluating sarcopenia, body composition, and both the quantity and quality of protein intake among institutionalized older adults in Ecuador remains scarce. Evaluating these factors simultaneously may improve the understanding of the nutritional and body composition characteristics associated with sarcopenia in this vulnerable population. In this context, the present study aimed to determine the prevalence of sarcopenia in institutionalized older adults (≥65 years), assess the adequacy of protein intake in terms of quantity and quality, analyze body composition, and identify factors associated with the presence of sarcopenia in this population.

## 2. Materials and Methods

### 2.1. Study Design and Setting

An observational, analytical, cross-sectional study was conducted between 2024 and 2025 in institutional care homes for older adults located in Loja canton, Ecuador.

### 2.2. Study Population and Sample

The study was carried out in three institutional care facilities for older adults. One facility was publicly funded and administered by the municipal government, whereas the other two were philanthropic non-profit institutions. At the time of the study, the three facilities housed a total of 108 residents, distributed as follows: 19 residents in the public institution and 41 and 48 residents in the two philanthropic institutions, respectively. All institutions provided medical care for their residents; however, none employed a nutritionist during the study period. In addition, nursing and physiotherapy students periodically provided support activities for residents as part of academic training programs.

**Figure 1:**
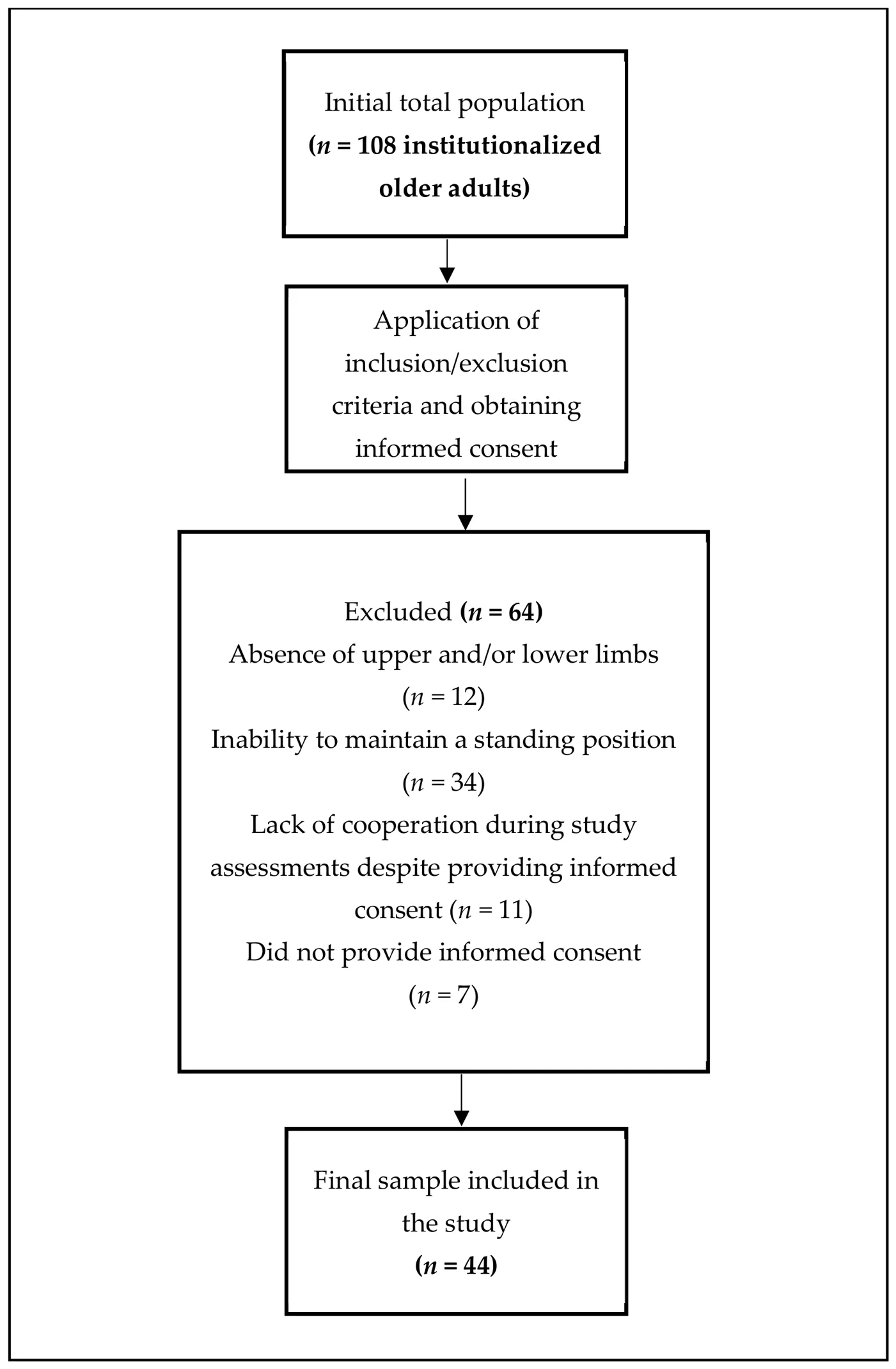
Flowchart of participant selection and inclusion in the study.

From a total population of 108 institutionalized individuals, after applying the inclusion and exclusion criteria and obtaining informed consent, a final sample of 44 participants was included (*n* = 44). The participant selection process is shown in Figure 1.

The study was approved by the Research Ethics Committee for Human Beings of the Universidad Técnica Particular de Loja (CEISH-UTPL) (approval code: 2024-07-INT-EO-RM-001). Ethical approval was granted on 2 October 2024 during an ordinary meeting of the CEISH-UTPL, and the official approval letter was issued on 3 October 2024. All participants provided written informed consent prior to participation.

### 2.3. Inclusion and Exclusion Criteria

Participants aged 65 years or older, of both sexes, with active enrollment in the institutions, without physical disabilities involving the absence of upper or lower limbs, and who provided informed consent were included. Individuals with edema, metallic implants, or uncontrolled endocrinopathies were excluded.

### 2.4. Variables

The study included demographic, clinical, functional, anthropometric, and nutritional variables.

- Demographic variables: Age, sex, and educational level.
- Clinical variable: Presence of sarcopenia.
- Functional variables: Muscle strength and physical performance.
- Anthropometric variables: Height, knee height, body weight, body mass index (BMI), appendicular skeletal muscle mass (ASM), ASM adjusted for height squared (ASM/height^2^ or skeletal muscle index), and body fat mass.
- Nutritional variables: Protein intake (quantity), protein quality (based on biological value), and total daily energy intake.

### 2.5. Data Collection and Procedures

#### 2.5.1. Sarcopenia Assessment

Sarcopenia was diagnosed according to the 2019 criteria of the European Working Group on Sarcopenia in Older People (EWGSOP2). It was classified as follows:

- Probable sarcopenia: Low handgrip strength (<27 kg in men, <16 kg in women).
- Confirmed sarcopenia: Low muscle strength plus low muscle mass (ASM/height^2^ <7 kg/m^2^ in men, <5.5 kg/m^2^ in women).
- Severe sarcopenia: Low muscle strength and muscle mass plus poor physical performance (Timed Up and Go ≥ 20 s) [11,12].

#### 2.5.2. Muscle Strength Assessment

Muscle strength was assessed using a CAMRY EH101 hand dynamometer (Zhongshan Camry Electronic Co., Ltd., Zhongshan, China). Participants were seated with proper posture (back supported, feet flat on the floor, elbow at 90°, wrist in a neutral position, and thumbs facing upward, with the arm supported). Three measurements were taken using the dominant hand, and the highest value was recorded [13].

#### 2.5.3. Body Composition Assessment

For muscle mass assessment, height was initially measured using a portable stadiometer (SECA 217, SECA GmbH & Co. KG, Hamburg, Germany). When direct measurement was not feasible, height was estimated using knee height measured with a CESCORF anthropometer (CESCORF Equipamentos para Esporte Ltda., Porto Alegre, Brazil) and Chumlea equations adjusted by sex and age [10]. Body composition, including ASM and ASM/height^2^, was assessed using a bioelectrical impedance analyzer (InBody 120, InBody Co., Ltd., Seoul, Republic of Korea). Bioelectrical impedance analysis (BIA) is widely used due to its simplicity, non-invasive nature, and accessibility compared to reference methods such as computed tomography, magnetic resonance imaging, and dual-energy X-ray absorptiometry [14].

#### 2.5.4. Physical Performance Assessment

Physical performance was evaluated using the Timed Up and Go (TUG) test, which measures the time required for the participant to stand up from a chair, walk three meters, turn around, return, and sit down again [11,15].

#### 2.5.5. Protein Intake Assessment

Protein intake was assessed using the direct food-weighing method to estimate the quantity and quality of dietary protein consumed. Foods were weighed before serving and leftovers were weighed after each meal using a CAMRY digital scale (Zhongshan Camry Electronic Co., Ltd., Zhongshan, Guangdong, China) over three non-consecutive days (two weekdays and one weekend day). All meals provided by the institutions were included. Actual food consumption for each participant was calculated as the difference between the amount of food served and the leftovers recorded after each meal. This method provides an objective estimate of actual dietary intake and minimizes recall bias [16,17].

The weight (g) of each food actually consumed was converted into nutrient intake using the Ecuadorian Food Composition Table (2021) and the Central American Food Composition Table (2012), which report nutrient composition per 100 g of edible portion. Protein intake from each food was calculated by multiplying the amount consumed (g) by its protein content per 100 g of edible portion, as reported in the food composition tables. Total daily protein intake (g/day) was obtained by summing the protein contribution of all foods consumed throughout the day. Daily energy intake was estimated using the same food composition tables.

Protein adequacy was assessed by comparing each participant’s estimated daily protein intake with current recommendations for older adults. A minimum intake of 1.0 g/kg body weight/day was considered adequate for healthy older adults, whereas higher recommendations (1.2–1.5 g/kg/day) were applied to participants with sarcopenia, according to current clinical guidelines [5]. Estimated energy requirements were calculated using the Harris–Benedict equation to assess energy adequacy (±10%) [10].

#### 2.5.6. Protein Quality Assessment

Protein quality was assessed using biological value tables, which reflect the efficiency with which ingested protein can be utilized for endogenous protein synthesis, considering both essential amino acid profile and bioavailability [17].

### 2.6. Statistical Analysis

Statistical analyses were performed using IBM SPSS Statistics for Windows, version 27 (IBM Corp., Armonk, NY, USA). Quantitative variables were assessed for normality using the Shapiro–Wilk test. Normally distributed variables were expressed as mean ± standard deviation (SD), while categorical variables were presented as frequencies and percentages.

Comparisons of quantitative variables between two groups (older adults with and without sarcopenia) were performed using the independent samples *t*-test, stratified by sex due to biological and functional differences between men and women. Effect size was calculated using Hedges’ g and interpreted as small (0.2), moderate (0.5), and large (≥0.8).

Associations between categorical variables were analyzed using the Chi-square test. When assumptions were not met due to low expected frequencies, Fisher’s exact test was applied.

To identify factors associated with sarcopenia, a bivariate binary logistic regression analysis was first performed, obtaining crude odds ratios (ORs) with 95% confidence intervals (95% CIs). Subsequently, a multivariate binary logistic regression model was constructed with sarcopenia as the dependent variable. Candidate variables included age, body mass index (BMI), body fat mass, skeletal muscle mass, and protein quality.

For the logistic regression analysis, sarcopenia status was dichotomized. Participants with confirmed or severe sarcopenia according to EWGSOP2 criteria were classified as the “sarcopenia” group, whereas those with probable sarcopenia and those without sarcopenia were classified as the “non-sarcopenia” group. This categorization was adopted to ensure sufficient sample size for a stable binary logistic regression model.

Collinearity was assessed using the variance inflation factor (VIF), and variables with VIF > 10 were excluded from the final model. The final adjusted model included age, BMI, skeletal muscle mass, and protein quality. All analyses were performed using IBM SPSS Statistics for Windows version 27, with statistical significance set at *p* < 0.05.

## 3. Results

### 3.1. Participant Characteristics

The baseline characteristics of the participants (*n* = 44) are summarized in Table 1. Comparative data are presented according to sex: males (*n* = 17) and females (*n* = 27).

The mean age was similar between groups (80 vs. 79 years). Regarding body composition, men showed significantly higher skeletal muscle mass (23.28 vs. 16.19 kg, *p* < 0.001) and skeletal muscle index (6.49 vs. 5.58 kg/m^2^, *p* = 0.006) compared with women. In terms of functional capacity, men also exhibited significantly greater muscle strength than women (14.09 vs. 9.55 kg, *p* = 0.005).

A higher proportion of men followed a normocaloric diet compared to women (35% vs. 3.7%, *p* = 0.005), although the predominant dietary pattern in both groups was hypercaloric. Regarding protein intake, both groups predominantly exhibited a hypoproteic diet (76.5% vs. 55.6%). No cases of low protein quality were identified in either group.

### 3.2. Prevalence of Sarcopenia

Table 2 shows the distribution of sarcopenia stages according to sex.

The overall prevalence of confirmed and severe sarcopenia in the study population was 61.3%, while 36.4% of participants had probable sarcopenia and 2.3% did not have sarcopenia.

Although no statistically significant differences were observed between sexes, a higher proportion of severe sarcopenia was identified in men (47.1% vs. 33.3%), whereas probable sarcopenia was more frequent in women (40.7% vs. 29.4%).

### 3.3. Comparison According to Sarcopenia Status in Men

Table 3 presents the characteristics of male participants according to sarcopenia status.

Participants with sarcopenia showed significantly lower skeletal muscle mass (26.61 vs. 21.46 kg, *p* < 0.001; g = 2.222) and skeletal muscle index (7.19 vs. 6.12 kg/m^2^, *p* = 0.001; g = 1.982), both with a large effect size.

Although body mass index (BMI) and body fat mass were lower in participants with sarcopenia, these differences were not statistically significant.

Regarding functional capacity, muscle strength was significantly lower in participants with sarcopenia (18.61 vs. 11.62 kg; *p* = 0.037; g = 1.104), with a large effect size. The mean time to complete the Timed Up and Go (TUG) test was higher in this group, indicating poorer physical performance, although the difference was not statistically significant.

In terms of protein intake, the amount of protein consumed was similar between groups (66.87 vs. 66.17 g/day; *p* = 0.904). However, participants with sarcopenia had significantly higher estimated protein requirements (87.67 vs. 72.63 g/day; *p* = 0.001; g = −2.112), resulting in lower protein adequacy (76.14% vs. 92.58%), although this difference did not reach statistical significance (*p* = 0.058). Protein quality was predominantly classified as high or very high in both groups, with no significant differences.

### 3.4. Comparison According to Sarcopenia Status in Women

Table 4 presents the characteristics of female participants according to sarcopenia status.

Body composition indicators, including BMI (29.58 vs. 23.73 kg/m^2^, *p* = 0.001; g = 1.419), skeletal muscle mass (18.23 vs. 14.78 kg, *p* = 0.001; g = 1.386), skeletal muscle index (6.62 vs. 4.87 kg/m^2^, *p* < 0.001; g = 2.123), and body fat mass (23.94 vs. 16.23 kg, *p* = 0.007; g = 1.106), were significantly higher in women without sarcopenia, with a large effect size.

No statistically significant differences were observed in muscle strength or physical performance (TUG), although women with sarcopenia showed lower strength and longer completion times.

Regarding protein intake, the amount of protein consumed was similar between women with and without sarcopenia (60.16 vs. 57.32 g/day; *p* = 0.470). However, women with sarcopenia had significantly higher estimated protein requirements (65.26 vs. 57.42 g/day; *p* = 0.025; g = −0.907), resulting in significantly lower protein adequacy (89.60% vs. 104.69%; *p* = 0.030; g = 0.873). Protein quality was mainly classified as moderate to very high in both groups, with no significant differences.

### 3.5. Protein Intake According to Sarcopenia Status

**Figure 2:**
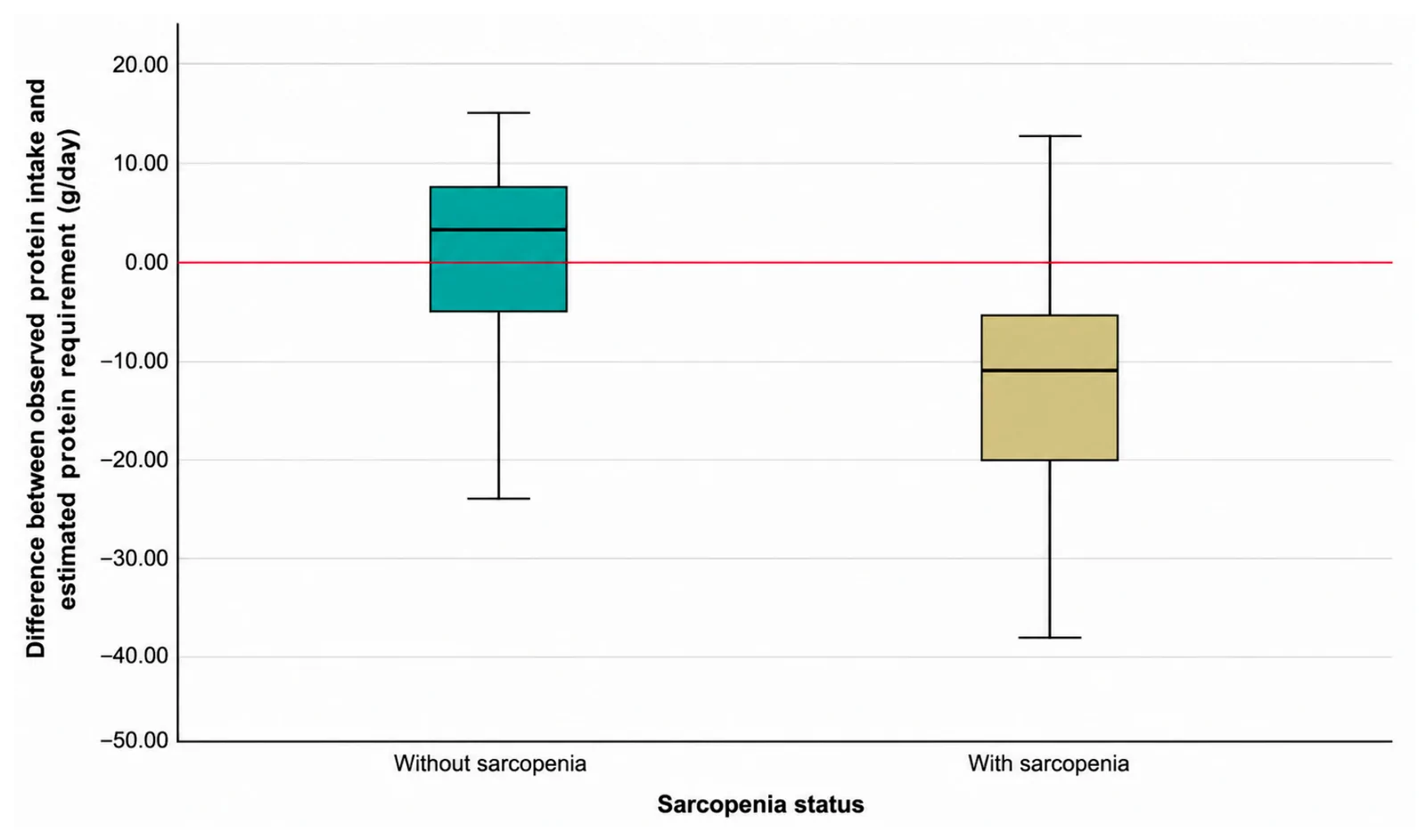
Difference between observed protein intake and estimated protein requirement according to sarcopenia status.

Figure 2 illustrates the distribution of the difference between observed protein intake and estimated protein requirements according to sarcopenia status.

Participants without sarcopenia showed median values close to zero, indicating that their observed protein intake was generally consistent with the estimated protein requirement based on current recommendations (1.0 g/kg/day). In contrast, participants with sarcopenia exhibited more negative values, indicating that their observed protein intake was insufficient relative to their higher estimated protein requirements (1.2–1.5 g/kg/day).

Greater variability and the presence of outliers were observed among participants with sarcopenia, suggesting greater heterogeneity in meeting their estimated protein requirements.

### 3.6. Factors Associated with Sarcopenia

Table 5 presents the results of the binary logistic regression analysis.

In the bivariate analysis, body fat mass, skeletal muscle mass, BMI, and protein quality were significantly associated with sarcopenia. Due to high collinearity (VIF > 10), body fat mass was excluded from the multivariate model. In the adjusted multivariate model, skeletal muscle mass and BMI remained independently associated with sarcopenia.

Higher skeletal muscle mass (adjusted OR = 0.771; 95% CI: 0.622–0.954; *p* = 0.017) and higher BMI (adjusted OR = 0.684; 95% CI: 0.517–0.907; *p* = 0.008) were associated with a lower likelihood of sarcopenia.

Age and protein quality were not significantly associated with sarcopenia in the adjusted model.

## 4. Discussion

The aim of this study was to investigate the prevalence of sarcopenia and its associated risk factors in older adults residing in three institutional care homes in Loja, Ecuador. In the study population (*n* = 44; mean age 80.11 years; 61.4% women), a high prevalence of sarcopenia (61.3%) was identified, which is consistent with previous studies conducted in institutionalized populations, where prevalence rates exceed 50% [18,19]. However, lower prevalence rates have also been reported, with studies in nursing home residents showing a prevalence of around 33.6%, highlighting variability depending on population characteristics and diagnostic criteria [20]. This variability has been widely described in the literature, with reported prevalence ranging from 17.7% to over 73.3% in long-term care settings [21].

Men showed a higher frequency of severe sarcopenia (47.1%), which aligns with studies reporting greater severity among institutionalized males [19,22]. In contrast, women exhibited a higher proportion of probable sarcopenia (40.7%), consistent with studies in which a large proportion of the population consisted of women presenting with early-stage sarcopenia [23].

Regarding nutritional status, both groups presented with mean BMI values within the normal range (25.21 kg/m^2^ in men and 26.11 kg/m^2^ in women), with no statistically significant differences. However, significant differences were observed in muscle mass and strength indicators, particularly in men with sarcopenia, who showed lower skeletal muscle mass, skeletal muscle index, and muscle strength. These findings are consistent with the EWGSOP2 criteria, which define reduced muscle mass and strength as key components in the diagnosis of sarcopenia [14]. This suggests that, despite BMI remaining within normal ranges, body composition and muscle function were the variables that differentiated older adults with sarcopenia from those without the condition. This is consistent with previous studies conducted in institutionalized older adults, where sarcopenia has been associated with poorer nutritional status, lower body mass index, reduced functional capacity, and a higher risk in individuals with greater functional dependence [24].

In both men and women, statistically significant differences in body composition indicators were observed between participants with and without sarcopenia. Among men, skeletal muscle mass and skeletal muscle index were significantly lower in those with sarcopenia (*p* < 0.001 and *p* = 0.001, respectively), along with a significant reduction in muscle strength (*p* = 0.037), all with large effect sizes, confirming functional loss associated with morphological deterioration. As reported in recent studies, physical performance in older adults is more strongly associated with muscle strength, whereas the relationship between muscle mass and physical performance may be weaker or more variable [25]. This reinforces the importance of muscle strength assessment as a key component in sarcopenia diagnosis.

In women, although no statistically significant differences were observed in muscle strength or physical performance, those with sarcopenia presented with significantly lower BMI, skeletal muscle mass, skeletal muscle index, and body fat mass (*p* = 0.001; *p* = 0.001; *p* < 0.01; and *p* = 0.007, respectively). These findings suggest that, in this group, sarcopenia may initially manifest through morphological and metabolic alterations rather than functional decline. Similar results have been reported in previous studies, where a higher prevalence of probable sarcopenia was observed in institutionalized women, characterized by reduced muscle mass without evident functional impairment [24].

Overall, the effect sizes observed in body composition indicators were large in both men and women, indicating that the differences between older adults with and without sarcopenia were not only statistically significant but also clinically relevant. This highlights the importance of body composition assessment in the detection and management of sarcopenia in institutionalized older adults, as BMI alone does not differentiate between muscle mass and fat mass and may underestimate the presence of sarcopenia. Therefore, international guidelines recommend the evaluation of muscle mass, muscle strength, and physical performance as part of the diagnosis and monitoring of this condition [13,15].

Sex-related differences observed in this study are consistent with previous findings suggesting that sarcopenia follows different trajectories in men and women, possibly due to hormonal, functional, and fat distribution differences. A study conducted in women aged 45 to 79 years reported a greater decline in bone mineral content compared to men, mainly attributed to postmenopausal estrogen deficiency [26]. The same study also described a progressive decline in muscle mass during the postmenopausal period [26].

Regarding protein intake, both men and women, with and without sarcopenia, showed intake levels below international recommendations (1.0–1.5 g/kg/day) [3,5]. This finding is relevant, as insufficient protein intake has been associated with the development and progression of sarcopenia in multiple studies [4,6,27]. In line with this, a recent meta-analysis reported that protein intake below 0.8 g/kg/day is associated with a higher risk of sarcopenia and reduced muscle strength, although no consistent associations were observed with muscle mass or physical performance indicators [28]. However, Figure 2 shows that participants without sarcopenia had protein intakes closer to recommended values, whereas those with sarcopenia exhibited greater variability and more pronounced deficits relative to recommended intake levels. Additionally, observational studies suggest that protein intake within the range of 1.0–1.6 g/kg/day is primarily associated with improvements in muscle strength and function rather than increases in muscle mass [8].

Although protein intake was quantified based on actual food consumption using the direct food-weighing method, the present study was not designed to determine whether the observed protein deficit was primarily attributable to insufficient protein provision by the institutions or to reduced dietary intake by residents. Because food portions were weighed before serving and leftovers were recorded after each meal, actual consumption could be estimated; however, the nutritional composition of institutional menus was not evaluated separately. In addition, factors such as poor appetite, chewing or swallowing difficulties, age-related anorexia, medication use, and other clinical conditions were not assessed and may have contributed to the low protein intake observed [5,29]. Therefore, the findings should be interpreted as reflecting the actual protein intake of residents in the participating institutions rather than allowing conclusions regarding the specific causes of inadequate protein consumption.

These findings support the existing literature, highlighting the importance of combined nutritional and physical activity interventions as the primary non-pharmacological strategy for preventing, attenuating, or even reversing sarcopenia in older adults [30,31]. Protein supplementation, with or without exercise, has been shown to improve muscle mass, strength, and physical function in older adults with sarcopenia and pre-frailty [32]. Furthermore, diets rich in high-biological-value proteins, such as those derived from animal sources or soy, may contribute to maintaining muscle mass in this vulnerable population [32].

According to current evidence, both the quantity and quality of dietary protein should be considered to reduce the risk of sarcopenia [33]. Observational studies suggest that animal-based proteins may have a more favorable effect on sarcopenia-related parameters compared to plant-based proteins [8]. It has also been proposed that sarcopenia may be mitigated through regular consumption of high-quality protein in adequate amounts [30].

Despite this evidence, no diets of low protein quality were identified in the present study, as all were classified as moderate, high, or very high in biological value. However, the prevalence of probable, confirmed, and severe sarcopenia remained high, suggesting that factors such as protein distribution throughout the day, total energy intake, comorbidities, and physical activity levels may have influenced the results [5,34,35].

Another factor that may contribute to musculoskeletal impairment in institutionalized older adults is vitamin D status. Vitamin D plays an important role in skeletal muscle physiology by regulating calcium metabolism, promoting protein synthesis, and supporting muscle cell differentiation and function [36]. Previous studies have reported associations between low serum 25-hydroxyvitamin D concentrations and adverse musculoskeletal outcomes in older adults, including osteoarthritis-related pain and functional limitations [37]. Furthermore, vitamin D deficiency has been recognized as a potential contributor to age-related muscle weakness and functional decline, particularly in older populations at increased risk of deficiency due to limited sun exposure and multiple comorbidities [36]. Although vitamin D status was not assessed in the present study, future research should consider its evaluation as a potential factor associated with sarcopenia in institutionalized older adults.

In the multivariate logistic regression analysis, skeletal muscle mass, and BMI were independently associated with sarcopenia. This suggests that, in this population, sarcopenia is primarily associated with body composition indicators. This finding may be explained by the fact that insufficient protein intake was a common characteristic among most participants, regardless of sarcopenia status, limiting its ability to differentiate between groups, whereas body composition variables showed stronger associations with sarcopenia. Higher skeletal muscle mass and higher BMI were associated with a lower likelihood of sarcopenia, reinforcing the importance of preserving muscle mass and maintaining adequate nutritional status in institutionalized older adults.

Although body fat mass was significantly associated with sarcopenia in the bivariate analysis, it was excluded from the multivariate model because of high collinearity (VIF > 10). Therefore, its independent contribution to sarcopenia could not be determined in the adjusted analysis. Future studies with larger sample sizes should further explore the potential role of adiposity and sarcopenic obesity in institutionalized older adults.

These findings should be interpreted with caution given the limited sample size and the exploratory nature of the multivariable analysis. Although the final model was simplified after assessment of collinearity, the number of events per variable was below the conventional threshold recommended for logistic regression models, which may affect the stability of the adjusted odds ratio estimates.

Finally, the findings of this study suggest that sarcopenia in institutionalized older adults is a multifactorial condition that depends not only on protein intake but also on overall nutritional status and body composition. Factors such as physical activity level, total energy intake, and comorbidities may also play a role in its development. Accordingly, a comprehensive approach to the prevention and management of sarcopenia should include adequate protein intake together with interventions aimed at preserving muscle mass, maintaining a healthy body composition, and implementing tailored physical exercise programs.

### Limitations and Strengths

This study has limitations related to its observational and cross-sectional design, which precludes establishing causal relationships between protein intake and sarcopenia. The sample size was relatively small and limited to three institutional care homes within a single canton, restricting the generalizability of the findings. Moreover, the limited sample size may have reduced the statistical power to detect differences between groups, particularly for variables that showed trends without reaching statistical significance. Therefore, the findings should be interpreted with caution and confirmed in larger multicenter studies involving more diverse institutionalized populations. In addition, detailed sociodemographic and clinical information was not available for individuals excluded during the participant selection process. Consequently, comparisons between included and excluded participants could not be performed and the potential for selection bias cannot be entirely excluded.

Additionally, information regarding nutritional and functional status prior to institutionalization was not available, limiting the ability to assess its influence on sarcopenia development or progression. Information regarding cognitive status, level of functional dependence, and participation in structured physical activity programs was not collected. Therefore, the potential influence of these factors on sarcopenia prevalence and severity could not be evaluated.

Although dietary intake was evaluated in terms of protein quantity and quality, the lack of data on prior dietary patterns, protein distribution throughout the day, and comorbidities limits a comprehensive interpretation of the findings. Moreover, factors potentially influencing dietary intake, such as appetite, chewing and swallowing difficulties, medication use, and the nutritional composition of institutional menus, were not assessed; therefore, the underlying causes of the observed protein deficit could not be determined.

Participants with probable sarcopenia were grouped together with those without sarcopenia for the binary logistic regression analysis, which may have limited the ability to distinguish between early-stage sarcopenia and the complete absence of the condition. Furthermore, although multivariable logistic regression was performed to explore factors associated with sarcopenia, the relatively small sample size resulted in a number of events per variable below the conventional recommendation for logistic regression models. Therefore, the adjusted odds ratios should be interpreted with caution, as the model may be susceptible to overfitting and reduced estimate stability.

Among the strengths, the use of the direct food-weighing allowed for an objective estimation of protein intake in terms of both quantity and quality. Additionally, internationally recognized diagnostic criteria (EWGSOP2) were used for the assessment of body composition and functional capacity. The statistical analysis included effect size estimation and adjusted logistic regression models, enabling the identification of both the magnitude of differences and factors associated with sarcopenia. Finally, this study provides local evidence on institutionalized older adults in Ecuador, a population that has been scarcely studied, contributing to the field of geriatric and community nutrition.

## 5. Conclusions

This study identified a high prevalence of sarcopenia among institutionalized older adults in Loja canton, with relevant differences in body composition and functional capacity between men and women. Protein intake was generally insufficient in both sexes, although protein quality did not show significant deficiencies based on biological value.

Higher skeletal muscle mass and higher body mass index were independently associated with a lower likelihood of sarcopenia. These findings suggest that sarcopenia in institutionalized older adults may be more closely related to body composition and overall nutritional status than to protein quantity or quality alone.

Preventive and management strategies for sarcopenia in this population may benefit from a comprehensive approach that includes adequate protein intake together with interventions aimed at preserving muscle mass, maintaining a healthy body composition, and implementing tailored physical exercise programs. Future studies with longitudinal designs and larger sample sizes are needed to better understand the factors associated with the development of sarcopenia in this population.

## Figures and Tables

**Table 1:** Sociodemographic, body composition, functional capacity, and dietary intake characteristics according to sex.

Variable	Category/Statistic	Sex		*p*-Value
		Men n (17)	Women n (27)	
**Sociodemographic indicators**				
Age (years)	Mean (SD)	80.35 (7.74)	79.96 (8.95)	0.883
Education level	No formal education F (%)	5 (29.4%)	3 (11.1%)	**0.007**
	Primary education F (%)	7 (41.2%)	4 (14.8%)	
	Secondary education F (%)	2 (11.8%)	3 (11.1%)	
	Higher education F (%)	3 (17.6%)	3 (11.1%)	
	Unknown F (%)	0 (0.0%)	14 (51.9%)	
**Body composition indicators**				
BMI (kg/m^2^)	Men (SD)	25.21 (3.16)	26.11 (4.89)	0.503
Skeletal muscle mass (kg)	Men (SD)	23.28 (3.31)	16.19 (2.92)	**<0.001**
Skeletal muscle index (kg/m^2^)	Men (SD)	6.49 (0.72)	5.58 (1.17)	**0.006**
Body fat mass (kg)	Men (SD)	20.07 (7.60)	19.37 (7.67)	0.771
**Functional capacity indicators**				
Muscle strength (kg)	Men (SD)	14.09 (6.75)	9.55 (5.05)	**0.015**
Physical performance (s)	Men (SD)	26.41 (16.64)	30.57 (21.41)	0.500
**Dietary intake indicators**				
Energy intake	Hypocaloric F (%)	0 (0.0%)	0 (0.0%)	**0.005**
	Normocaloric F (%)	6 (35.3%)	1 (3.7%)	
	Hypercaloric F (%)	11 (64.7%)	26 (96.3%)	
Protein intake	Low protein intake F (%)	13 (76.5%)	15 (55.6%)	0.057
	Adequate protein intake F (%)	3 (17.6%)	2 (7.4%)	
	High protein intake F (%)	1 (5.9%)	10 (37.0%)	
Protein quality	Moderate-quality F (%)	1 (5.9%)	9 (33.3%)	0.056
	High-quality F (%)	11 (64.7%)	9 (33.3%)	
	Very-high-quality F (%)	5 (29.4%)	9 (33.3%)	

Statistical analysis was performed using Pearson’s chi-square test. A *p*-value < 0.05 was considered statistically significant. Bold values indicate statistical significance. BMI: Body Mass Index.

**Table 2:** Distribution of sarcopenia in the study population.

		Sarcopenia				
		Probable Sarcopenia	Confirmed Sarcopenia	Severe Sarcopenia	No Sarcopenia	*p*-Value
**Sex**	Men F (%)	5 (29.4%)	3 (17.6%)	8 (47.1%)	1 (5.9%)	0.508
	Women F (%)	11 (40.7%)	7 (25.9%)	9 (33.3%)	0 (0.0%)	
Total	F (%)	16 (36.4%)	10 (22.7%)	17 (38.6%)	1 (2.3%)	

Statistical analysis was performed using Fisher’s exact test. A *p*-value < 0.05 was considered statistically significant.

**Table 3:** Comparison of demographic characteristics, body composition, functional capacity, and protein intake in older men according to the presence of sarcopenia, including mean differences, *p*-values, and effect size (Hedges’ g).

		Sarcopenia				
		No Sarcopenia	Sarcopenia	Mean Difference	*p*-Value	Hedges’ g
**Sociodemographic indicator**						
Age (years)	Mean (SD)	79.83 (8.49)	80.64 (7.72)	−0.803	0.846	−0.095
**Body composition indicators**						
BMI (kg/m^2^)	Mean (SD)	26.31 (3.78)	24.60 (2.77)	1.707	0.302	0.514
Skeletal muscle mass (kg)	Mean (SD)	26.61 (2.85)	21.46 (1.79)	5.153	**<0.001**	2.222
Skeletal muscle index (kg/m^2^)	Mean (SD)	7.19 (0.53)	6.12 (0.50)	1.068	**0.001**	1.982
Body fat mass (kg)	Mean (SD)	21.03 (9.43)	19.54 (6.87)	1.487	0.713	0.181
**Functional capacity indicators**						
Muscle strength (kg)	Mean (SD)	18.61 (7.09)	11.62 (5.38)	6.989	**0.037**	1.104
Physical performance (s)	Mean (SD)	17.98 (11.76)	31.00 (17.56)	−13.021	0.127	−0.779
**Protein intake indicators**						
Protein intake (g/day)	Mean (SD)	66.87 (12.52)	66.17 (10.43)	0.696	0.904	0.059
Recommended protein requirement (g/day)	Mean (SD)	72.63 (6.32)	87.67(6.95)	−15.027	0.001	−2.112
Protein adequacy (%)	Mean (SD)	92.58 (18.33)	76.14 (14.37)	16.443	**0.058**	0.987
Protein quality	Moderate-quality F (%)	0 (0.00)	1 (5.88)	0.599	0.741	
	High-quality F (%)	4 (23.53)	7 (41.18)			
	Very-high-quality F (%)	2 (11.76)	3 (17.65)			

Differences between groups (with and without sarcopenia) were assessed using the independent samples *t*-test. A *p*-value < 0.05 was considered statistically significant. Effect size was calculated using Hedges’ g and interpreted as small (0.2), moderate (0.5), and large (≥0.8). BMI: Body Mass Index.

**Table 4:** Comparison of demographic characteristics, body composition, functional capacity, and protein intake in older women according to the presence of sarcopenia, including mean differences, *p*-values, and effect size (Hedges’ g).

		Sarcopenia				
		No Sarcopenia	Sarcopenia	Mean Difference	*p*-Value	Hedges’ g
**Sociodemographic indicator**						
Age (years)	Mean (SD)	78.45 (9.44)	81.00 (8.75)	−2.545	0.479	−0.273
**Body composition indicators**						
BMI (kg/m^2^)	Mean (SD)	29.58 (4.47)	23.73 (3.64)	5.850	**0.001**	1.419
Skeletal muscle mass (kg)	Mean (SD)	18.23 (2.40)	14.78 (2.41)	3.448	**0.001**	1.386
Skeletal muscle index (kg/m^2^)	Mean (SD)	6.62 (0.74)	4.87 (0.83)	1.750	**<0.001**	2.123
Body fat mass (kg)	Mean (SD)	23.94 (8.83)	16.23 (4.90)	7.707	**0.007**	1.106
**Functional capacity indicators**						
Muscle strength (kg)	Mean (SD)	10.30 (6.51)	9.04 (3,90)	1.265	0.533	0.240
Physical performance (s)	Mean (SD)	29.48 (25.67)	31.31 (18.81)	−1.834	0.832	−0.082
**Protein intake indicators**						
Protein intake (g/day)	Mean (SD)	60.16 (11.60)	57.32 (6.95)	−8.420	0.470	0.303
Recommended protein requirement (g/day)	Mean (SD)	57.42 (6.39)	65.26 (9.49)	−7.841	0.025	−0.906
Protein adequacy (%)	Mean (SD)	104.69 (15.90)	89.60 (17.30)	15.096	**0.030**	0.873
Protein quality	Moderate-quality F (%)	5 (18.50)	4 (14.80)	1.227	0.541	
	High-quality F (%)	3 (11.10)	6 (22.20)			
	Very-high-quality F (%)	3 (11.10)	6 (22.20)			

Differences between groups (with and without sarcopenia) were assessed using the independent samples *t*-test. A *p*-value < 0.05 was considered statistically significant. Effect size was calculated using Hedges’ g and interpreted as small (0.2), moderate (0.5), and large (≥0.8). BMI: Body Mass Index.

**Table 5:** Factors associated with sarcopenia in institutionalized older adults using binary logistic regression.

			95% CI				95% CI	
	Crude OR	*p*-Value	Lower	Upper	Adjusted OR	*p*-Value	Lower	Upper
**Age (years)**	1.028	0.461	0.954	1.108	1.006	0.911	0.903	1.121
**Skeletal muscle mass (kg)**	0.365	**0.017**	0.160	0.833	0.771	0.017	0.622	0.954
**BMI (kg/m^2^)**	0.229	**0.010**	0.075	0.706	0.684	0.008	0.517	0.907
**Protein quality**	0.000	**0.043**	0.000	0.788	2.153	0.260	0.567	8.177

Results are presented as odds ratios (ORs) with 95% confidence intervals (95% CIs), obtained using binary logistic regression. The crude OR corresponds to the bivariate analysis, and the adjusted OR to the multivariate model. An OR > 1 indicates a higher probability of sarcopenia, whereas an OR < 1 indicates a lower probability. Statistical significance was set at *p* < 0.05. Body fat mass was excluded from the final multivariate model because of high collinearity (VIF > 10). Skeletal muscle index, muscle strength, physical performance, and protein intake were excluded because of their direct relationship with the diagnostic criteria for sarcopenia and to minimize model overadjustment.

## Data Availability

The raw data supporting the conclusions of this article will be made available by the authors on request.
